# Poor-Law Infirmaries as Training Schools

**Published:** 1913-09-13

**Authors:** G. J. Masson Martin

**Affiliations:** Senior A.M.O., Portsmouth Infirmary.


### POOR-LAW INFIRMARIES AS TRAINING SCHOOLS.

By G. J. MASSON MARTIN, L.R.C.P. & S. I., D.P.H., Senior A.M.O., Portsmouth Infirmary.

### II.—As Nurse Training Schools: A Comparison.*

THE suitability of Poor-Law infirmaries as training schools for nurses falls under quite a different category to that of the similar fitness of such institutions for doctors. In the latter case these infirmaries represent merely finishing schools where persons already qualified in their profession may have opportunities of perfecting themselves in their work before commencing practice. In the question now under discussion a much more serious object has to be attained—namely, the training of persons for a profession in the work of which they have no former knowledge, and, after such training is complete, the granting of a certificate which will prove them to be competent persons to be entrusted with the nursing of the sick.

Up to the present, unfortunately, no standard has been fixed as to what practical instruction must be given, what examinations must be passed, or what the length of instruction must be, to ensure this competency, and it speaks well for the medical and nursing professions at large, and the institutions which turn out so many nurses during the year, that such a high standard of efficiency has been reached, and is maintained.

Up to the present the only institutions where nurses are trained in which any attempt has been made at a standardisation of the course of instruction by a responsible body is in Poor-Law infirmaries. The Local Government Board has drawn up a set of rules to which such institutions must conform in the training of their nurses before they will be recognised as training schools and capable of granting a certificate of proficiency in nursing.

There is an unfortunate bias in the minds of a certain section of the public, and also, what is still more unfortunate, amongst certain members of the medical profession, that the training given in workhouse infirmaries is inferior to that given in the large hospitals—and indeed, any hospital, large or small. It is only the larger infirmaries, as in the former part of this article, which are indicated here; the smaller ones are not generally recognised by the Local Government Board as training schools. It will be found, as a rule, that this bias is met with only in those who are unacquainted with the efficiency of modern Poor-Law institutions, and is generally engendered by a mere association of ideas—the word workhouse when used in connection with infirmary suggesting poverty, dirt, and incompetence. One of the facts which goes to prove that such a bias is entirely unmerited is that mentioned above of the standardisation of the training in infirmaries by the Local Government Board—certain examinations must be passed, certain lectures must be given, and a definite period of practical ward work must be completed before a certificate can be obtained. Training in general hospitals is subject to no such restrictions—each hospital being a law unto itself—no lectures need be given, no examinations need be passed, and the period which must be spent in ward work varies in each institution.

There are other reasons also which go to show that Poor-Law infirmaries must present an equally good training as general hospitals. One of these reasons is that the nursing staff and their work in Poor-Law infirmaries is subject at any time to inspection and criticism by the Local Government Board inspectors. These officials are specially appointed for this purpose, their inspection and criticism being by no means lax, and if the training is, in their opinion, not up to the most modern standard and full and unimpeachable in every branch those responsible for such training are liable to very severe censure. The nursing staff in general hospitals is liable to no such official inspection and criticism.

Another reason is that in Poor-Law infirmaries senior nurses are expected to perform, besides their usual nursing duties, minor surgical and medical procedures, such as the removal of stitches and the passage of a stomach tube, duties which are in almost all cases carried out by house surgeons or students in general hospitals. They are also often allowed to assist at major operations, which again is a duty which is carried out by the junior male staff in hospitals. Nurses may be called upon at any time to perform these duties afterwards in private nursing practice. Greater experience in acute surgical work may be obtained by the hospital-trained nurse, but she will probably not be called upon to nurse many such cases after her training is complete—the majority will be cases of chronic disease which are so numerous in workhouse infirmaries.

It must not be thought that in making this comparison any effort is being made to decry in any way the training which is given by hospitals—on the contrary, the standard in all hospitals of repute is very high—but still it is only fair to endeavour to show that Poor-Law infirmary training is equally good, and that nurses so trained are equally competent to carry out any duties which may fall to the lot of a nurse as those who are trained elsewhere.

Another advantage which must not be overlooked is that the age limit for starting training is lower in infirmaries than in most general hospitals. Training may be obtained, too, in workhouse infirmaries without any preliminary monetary outlay. In conclusion, it may be stated that any woman of average intelligence who has been through a training period of three years in a modern large Poor-Law infirmary can feel thoroughly assured of her ability to fulfil satisfactorily any nursing duty she may be called upon to perform, and may be entrusted with confidence by any medical man as fitted to carry out the nursing of any disease with perfect efficiency.

* The article on Training Schools for Doctors appeared on July 19, 1913.

